# Modified PEG-Lipids Enhance the Nasal Mucosal Immune Capacity of Lipid Nanoparticle mRNA Vaccines

**DOI:** 10.3390/pharmaceutics16111423

**Published:** 2024-11-07

**Authors:** Meng Li, Jing Yi, Yicheng Lu, Ting Liu, Haonan Xing, Xiwei Wang, Hui Zhang, Nan Liu, Zengming Wang, Aiping Zheng

**Affiliations:** 1 Beijing Institute of Pharmacology and Toxicology, 27 Taiping Road, Haidian District, Beijing 100850, China; limeng854321@126.com (M.L.);; 2 College of Pharmacy, Yanbian University, 977 Park Road, Yanji 133002, China; 3 School of Pharmaceutical Sciences, Capital Medical University, 10 You’anmen Outer West 1st Street, Beijing 100069, China

**Keywords:** chitosan-modified PEGylated lipids, mannose-modified PEGylated lipids, lipid nanoparticles, mucosal immunity, mRNA vaccine

## Abstract

Background/Objectives: Omicron, the predominant variant of SARS-CoV-2, exhibits strong immune-evasive properties, leading to the reduced efficacy of existing vaccines. Consequently, the development of versatile vaccines is imperative. Intranasal mRNA vaccines offer convenient administration and have the potential to enhance mucosal immunity. However, delivering vaccines via the nasal mucosa requires overcoming complex physiological barriers. The aim of this study is to modify PEGylated lipids to enhance the mucosal immune efficacy of the vaccine. Methods: The PEGylated lipid component of lipid nanoparticle (LNP) delivery vectors was modified with chitosan or mannose to generate novel LNPs that enhance vaccine adhesion or targeting on mucosal surfaces. The impact of the mRNA encoding the receptor-binding domain of Omicron BA.4/BA.5 on the immune response was examined. Results: Compared to the unmodified LNP group, the IgG and IgA titers in the chitosan or mannose-modified LNP groups showed an increasing trend. The chitosan-modified group showed better effects. Notably, the PEGylated lipid with 1.5 mol% of chitosan modification produced high levels of IgG1 and IgG2a antibodies, promoting Th1/Th2 responses while also generating high levels of IgA, which can induce stronger cellular immunity, humoral immunity, and mucosal immunity. Conclusions: The 1.5 mol% of chitosan-modified LNPs (mRNA-LNP-1.5CS) can serve as a safe and effective carrier for intranasal mRNA vaccines, offering a promising strategy for combating the Omicron variant.

## 1. Introduction

Coronavirus disease 2019 (COVID-19) is caused by severe acute respiratory syndrome coronavirus 2 (SARS-CoV-2), a positive-sense single-stranded RNA virus within the β-coronavirus genus [1]. The first generation of mRNA- and adenovirus-vectored vaccines effectively mitigated the COVID-19 pandemic after licensure; however, they exhibited reduced efficacy against asymptomatic infections and did not prevent viral transmission [2]. The high mutation rate and genetic diversity of SARS-CoV-2 [3] present significant challenges in developing a universally effective vaccine. The emergence of numerous variants of concern (VOC) has also diminished the efficacy of existing vaccines [4,5,6]. However, mRNA vaccines, which have garnered significant attention, can be rapidly updated and iterated to cope with the continuous emergence of mutated strains [7]. SARS-CoV-2 primarily spreads via the respiratory tract, initiating infection in the nasal mucosa. Unlike natural infections, first-generation vaccines administered via the intramuscular route do not elicit mucosal IgA or respiratory T cell responses, nor do they prevent respiratory infections, particularly against VOC with multiple mutations in the Spike protein [8,9]. Omicron, the predominant variant of SARS-CoV-2, significantly reduces the neutralizing potency of different epitopes of neutralizing antibodies and exhibits robust immune-evasive capabilities. For example, infection with the Omicron BA.1 variant offers only 70–80% protection against infection with BA.4 or BA.5 [10]. Studies have demonstrated that reducing the nasal viral load, activating the nasal mucosa, and enhancing the self-cleaning ability and immune capacity of the nasal mucosa can effectively remove the virus and reduce the risk of SARS-CoV-2 transmission and infection [11,12]. Therefore, there is an urgent need to develop novel COVID-19 mRNA vaccines that can induce mucosal and local immunological memories. Intranasal immunization is an effective method to generate strong mucosal immunity against COVID-19 [13,14].

Intranasal vaccines, as opposed to traditional intramuscular injections, offer a needle-free and noninvasive method of immunization that is easier to implement, does not require injection by professional health personnel, and can be administered by trained personnel or possibly via self-administration in a universal immunization campaign [15], with better compliance and applicability. This is especially true during global outbreaks of infectious diseases, such as respiratory infections. Despite these advantages, intranasal mRNA vaccination faces the following obstacles: the presence of enzymes in the nasal cavity may degrade mRNA, and ciliary movement may accelerate the clearance of mRNA antigens and shorten their residence time in the nasal cavity, resulting in poor antigen uptake [16]. To ensure the successful delivery of intranasal mRNA vaccines, addressing these physiological complexities, including the nasal mucosal layer and tightly connected epithelial barriers, is crucial.

As negatively charged mRNA molecules cannot easily enter cells through negatively charged cell membranes and because of the special structure of the nasal cavity, naked mRNA molecules are easily degraded by plasma or tissue enzymes [16,17]. Efficient vaccine delivery systems need to be developed to address these challenges. Lipid nanoparticles (LNPs) are promising vectors for mRNA delivery [18]. Although LNPs are adept at delivering mRNA, the mucosal layer and epithelial barrier continue to pose a formidable challenge to the intranasal delivery of mRNA vaccines. Chitosan (CS) has the advantages of a strong positive charge, immune adjuvant effect, and enhanced antigen absorption. CS can be electrostatically adsorbed or hydrogen-bonded to mucin oligosaccharide chains to promote its adhesion to the mucosal surface, which is widely used in mucosal immunity [19,20,21,22,23]. Mannose (Man) receptors are highly expressed on the surface of dendritic cells (DCs) and macrophages. These receptors mediate endocytosis by DCs and activate DCs through the Toll-like receptor (TLR) signaling pathway and then present antigens to CD4^+^ and CD8^+^ T cells [24]. Multiple studies have reported that mannoglycated drug delivery systems targeting mucosal DC can significantly boost immune response [25,26,27]. Therefore, the modification of LNPs with CS or Man may be a strategy for improving the efficiency of intranasal mRNA vaccine delivery.

The structure of LNPs commonly includes ionizable lipids, phospholipids, cholesterol, and polyethylene glycol (PEG)-lipids, each of which plays a critical role in formulation [28]. Although PEG-lipids constitute the smallest molar percentage of lipid components in LNPs (typically ~1.5 mol%), they influence the particle size and distribution, encapsulation efficiency, in vivo distribution, transfection efficiency, and immune response [29,30]. The tail structure of PEG lipids also affects the biological activity of LNPs [28]. As PEG-lipids are incorporated into LNP membranes via hydrophobic tails (alkyl/acyl chains), PEG-lipids with longer tails are less likely to dissociate from LNPs [28]. The PEG-lipid alkyl chain used in commercially available COVID-19 mRNA-LNP vaccines consists of 14C (e.g., DMG-PEG2000 and ALC-0159), which exhibits a weaker binding force than the 18C structural phospholipids in LNPs (e.g., DSPE-PEG2000) [31] and easily dissociates in complex environments in vivo. Substituting slow-desorbing PEG-lipids with fast-desorbing PEG-lipids abrogates the strong immune response to PEGylated liposomes containing nucleic acid cargo [32]. Based on these properties, we hypothesized that LNPs composed of DMG-PEG2000 modified with CS or Man (DMG-PEG2000-CS or DMG-PEG2000-Man) may serve as effective carriers for nasal mRNA vaccine delivery.

In this study, the Omicron BA.4/BA.5 receptor-binding domain (RBD) was selected as the model antigen. A microfluidic device was used to prepare mRNA-LNPs with varying molar ratios of DMG-PEG2000-CS to DMG-PEG2000-Man. We then compared the efficacy of CS-modified- or Man-modified LNPs (i.e., LNP-CS or LNP-Man) at different molar ratios in delivering Omicron BA.4/BA.5 RBD mRNA and inducing specific immune responses. The transfection efficiency, endosomal escape, and cellular uptake mechanism of the delivery vectors were assessed in DC 2.4 cells. Additionally, activation of the costimulatory molecules CD40, CD80, and CD86 by delivery vectors was evaluated in bone marrow-derived dendritic cells (BMDCs). Finally, the immune response and biosafety of the mRNA complexes were investigated in a mouse model after intranasal immunization. This comprehensive evaluation aimed to elucidate the potential of these tailored LNPs as carriers of antigen-specific mRNA in intranasal vaccine delivery systems.

## 2. Materials and Methods

### 2.1. Materials

Plasmids encoding the gene sequences of the SARS-CoV-2 Spike RBD (Omicron BA.4/BA.5) and RBD-eGFP were synthesized by the Genscript Biotech Corporation (Nanjing, China). A T7 High Yield RNA Transcription kit, Vaccinia Capping Enzyme, and mRNA Cap 2′-O-Methyltransferase were purchased from Vazyme Biotech Co., Ltd. (Nanjing, China). Cy3-UTP was purchased from MedBio (Shanghai, China). SapI Restriction Endonuclease was purchased from New England Biolabs (Beverly, MA, USA). Citrate buffer (25 mM, pH 4.0) was purchased from Leagene Biotechnology Co., Ltd. (Beijing, China). 1,2-Dimyristoyl-rac-glycero-3-methoxypolyethylene glycol-2000 (DMG-PEG2000) and cholesterol were obtained from AVT (Shanghai, China) Pharmaceutical Technology Co., Ltd. (Shanghai, China). 1,2-distearoyl-sn-glycero-3-phosphocholine (DSPC) and 1-octylnonyl 8-[(2-hydroxyethyl)[6-oxo-6-(undecyloxy)hexyl]amino]octanoate (SM-102) were purchased from SINOPEG (Xiamen, China). DMG-PEG2000-Mannose (DMG-PEG2000-Man, MW ~2700) and DMG-PEG2000-Chitosan (DMG-PEG2000-CS, MW 4500-4600) were custom synthesized by Xi’an Ruixi Biological Technology Co., Ltd. (Xi’an, China). Nystatin, chlorpromazine hydrochloride, and ethylisopropylamiloride (EIPA) were purchased from Solarbio Life Sciences (Beijing, China). Methyl-β-Cyclodextrin (MβCD) was obtained from Zibo Qianhui Biological Technology Co., Ltd. (Zibo, China). LiCl, Hoechst 33258, and DiI were purchased from Beyotime Biotechnology (Shanghai, China). The Brilliant Violet 421^TM^ anti-mouse CD11c antibody, APC anti-mouse CD40 antibody, PE anti-mouse CD80 antibody, and Brilliant Violet 510^TM^ anti-mouse CD86 antibody were purchased from BioLegend (San Diego, CA, USA). Alexa Fluor^®^ 647 Anti-LAMP1 antibody, Goat Anti-Mouse IgG1 (HRP), and Goat Anti-Mouse IgG2a heavy chain (HRP) were purchased from Abcam (Cambridge, MA, USA). Dulbecco’s modified Eagle medium (DMEM) and fetal bovine serum (FBS) were purchased from HUANKE (Beijing, China). The RPMI 1640 medium was purchased from Wisent Corporation (Nanjing, China). Opti-MEM^®^ was purchased from GIBCO BRL (Grand Island, NY, USA). Goat Anti-Mouse IgA (α-chain specific)-Peroxidase was obtained from Sigma-Aldrich (Shanghai, China) Trading Co., Ltd. (Shanghai, China).

### 2.2. Methods

#### 2.2.1. mRNA Preparation and Identification

**Figure 1:**
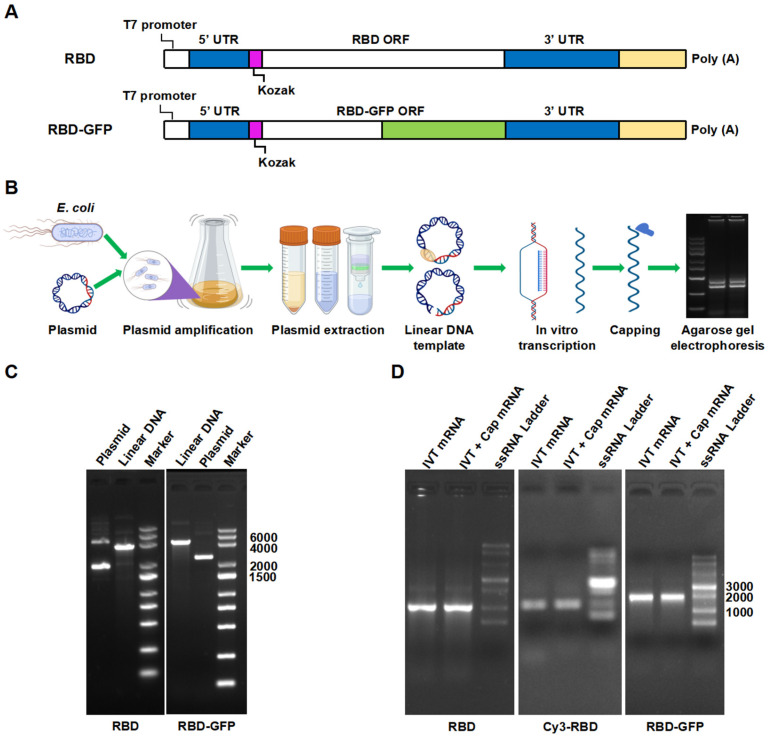
(**A**) Schematic diagram of mRNA gene sequence. (**B**) Schematic overview of the mRNA production process. (**C**) Linear DNA agarose gel electrophoresis. (**D**) mRNA agarose gel electrophoresis.

The plasmid encoding the target gene sequence (Figure 1A) was isolated from *Escherichia coli* (*E. coli*). Plasmids encoding the RBD and RBD-eGFP fusion proteins were linearized using SapI restriction endonuclease and subsequently purified via phenol extraction. The mRNA was produced by in vitro transcription and capped following the protocols provided with the T7 High Yield RNA Transcription kit, Vaccinia Capping Enzyme, and mRNA Cap 2′-O-Methyltransferase before being purified using lithium chloride. For preparing the Cy3-labeled mRNA, 25% of the UTP was substituted with Cy3-UTP, and the remaining preparation steps were aligned with those used for RBD mRNA. The integrity of DNA and mRNA was confirmed using agarose gel electrophoresis. A schematic representation of the mRNA production process is shown in Figure 1B.

#### 2.2.2. LNP Formulation and Characterization

**Figure 2:**
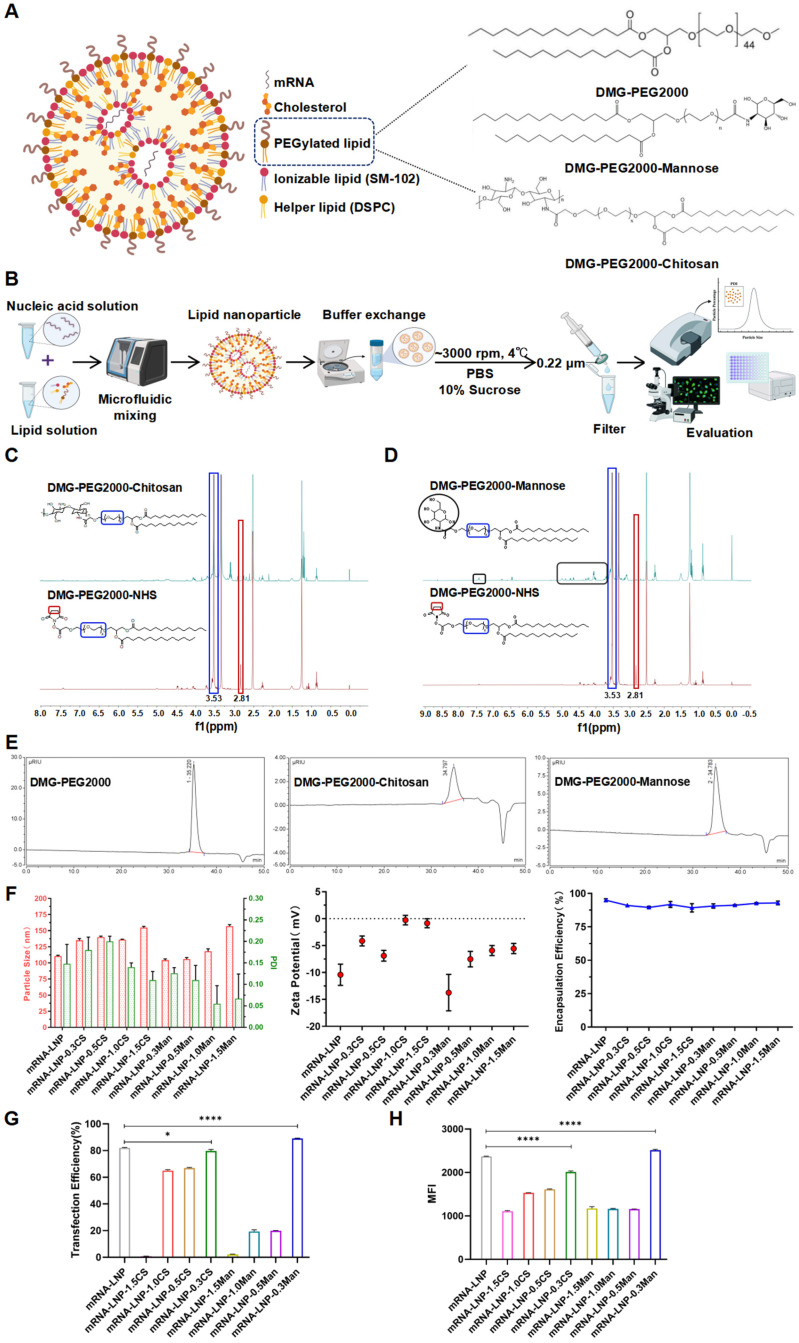
(**A**) Schematic illustration of LNPs and PEGylated lipid structures. (**B**) Development and characterization flow chart of LNPs. (**C**) ^1^H-NMR of DMG-PEG2000-NHS and DMG-PEG2000-Chitosan. (**D**) ^1^H-NMR of DMG-PEG2000-NHS and DMG-PEG2000-Mannose. (**E**) Graphs of GPC for Different PEGylated Lipids. (**F**) Particle size, polydispersity index (PDI), zeta potential, and encapsulation efficiency of LNPs. (**G**) Transfection efficiency of LNPs in DC2.4 cells. (**H**) Mean fluorescence intensity (MFI) analysis of different LNPs assessed by Flow CytometryTypical flow cytometry (FCM). * *p* < 0.05, **** *p* < 0.0001.

LNPs were prepared via microfluidic mixing using a previously reported method [33]. Lipid-ethanol solutions containing SM-102, cholesterol, DSPC, DMG-PEG2000, DMG-PEG2000-Man, or DMG-PEG2000-CS were mixed with a 25 mM citrate buffer (pH 4.0) containing mRNA at a 1:3 ratio within a microfluidic mixer (Figure 2A,B). The molar ratios of different lipid components and other procedural parameters are listed in Table 1. The LNPs were eluted twice with five times the volume of phosphate-buffered saline (PBS, pH 7.4), centrifuged at 4 °C (~3000 rpm) using a 100 kDa MWCO (Millipore, Billerica, MA, USA) centrifugal filter, eluted and concentrated with five times the volume of PBS solution containing 10% sucrose, and finally filtered through a 0.22 μm filter membrane. A schematic representation of the overall process is shown in Figure 2B. DiI-labeled LNPs (DiI-LNPs) were prepared by adding an appropriate DiI solution (final concentration: 10 μg/mL) to the LNPs, avoiding light, and incubating at room temperature for 10 min. The remaining steps followed the same procedure used for LNP preparation. The size distribution and zeta potential of the LNPs were determined via dynamic light scattering using a Malvern Zetasizer Nano ZS90 (Malvern Instruments, Malvern, UK). The mRNA encapsulation efficiency was quantified using the Quant-iT RiboGreen RNA reagent kit (Invitrogen, Carlsbad, CA, USA), to provide an assessment of the mRNA encapsulated within the LNPs.

#### 2.2.3. Transfection Efficiency of LNPs in DC2.4 Cells

To study the transfection efficiency of various LNP formulations, DC2.4 cells were seeded in 12-well plates at a density of 5 × 10^5^ cells/well. Each well was supplemented with 1.0 mL of DMEM containing 10% FBS and 1% of a Penicillin-Streptomycin-Amphotericin B Solution. The cells were cultured at 37 °C in a 5% CO_2_ atmosphere for 24 h. The medium was discarded when cells reached approximately 70% confluence. The samples were then diluted with 1.0 mL/well of low serum Opti-MEM^®^ containing 1 μg of RBD-eGFP-mRNA and incubated for 6 h. Subsequently, the Opti-MEM^®^ was aspirated and replaced with 1.0 mL of fresh DMEM, and the cells were cultured for an additional 24 h. After the incubation period, the medium was discarded, and 200 μL of 0.25% Trypsin-EDTA was added to each well to enzymatically digest the cells for 2 min. The digestion process was terminated by adding 1 mL of complete DMEM, followed by gentle agitation to ensure thorough suspension of cells. The resulting cell suspension was transferred into a 1.5 mL centrifuge tube and centrifuged at 350× *g* for 5 min at 4 °C. The supernatants were carefully aspirated. Cells were washed twice with PBS and resuspended in PBS (0.5 mL). Transfection efficiencies of different LNP formulations were evaluated using an Attune NxT flow cytometry analyzer (Invitrogen, Carlsbad, CA, USA).

#### 2.2.4. Morphological Characterization of LNPs Using Transmission Electron Microscopy (TEM)

The morphology of LNPs was characterized using TEM. A 10 μL aliquot of the sample solution was placed onto carbon-coated copper grids and allowed to dry at 25 °C. Subsequently, the grids were stained with 0.1% (*w*/*v*) phosphotungstic acid for 2 min and blotted onto filter paper. After air-drying, the morphology of the nanoparticles was observed using TEM (H-7650, HITACHI, Tokyo, Japan). The accelerating voltage was set to 80 kV.

#### 2.2.5. Cellular Uptake

To investigate the endocytic pathways of LNPs, DC 2.4 cells were seeded in 12-well plates at a density of 5 × 10^5^ cells/well and cultured for 24 h at 37 °C with 5% CO_2_. After incubation, the culture medium was discarded, and the cells were gently washed twice with PBS. Subsequently, inhibitor solutions (0.5 mL) at varying concentrations diluted in Opti-MEM^®^ were added to each well and incubated for 30 min. Three wells were designated for each inhibitor compound. The functions and concentrations of the inhibitors are listed in Table 2. The solutions were discarded following incubation with inhibitors, and 1 mL of DiI-LNPs, diluted in the corresponding inhibitor solution, were added to each well, with an mRNA concentration of 1 μg/mL. The cells were incubated for an additional 6 h. Opti-MEM^®^ without inhibitors served as the positive control, while Opti-MEM^®^ without inhibitors and DiI-LNPs served as the negative control. After incubation, the samples were processed under light-avoiding conditions, following the same procedure as in the transfection efficiency experiments. The cellular uptake of LNPs in the presence of different inhibitors was quantified using an Attune NxT flow cytometry analyzer.

#### 2.2.6. Endosome Escape

DC2.4 cells were seeded in confocal dishes at a density of 1 × 10^5^ cells/well and incubated for 36 h in an incubator at 37 °C with 5% CO_2_. After incubation, the original medium was discarded, and LNPs containing Cy3-mRNA (mRNA concentration was 1 μg/mL) were added to the confocal dishes. Simultaneously, 1 mL of Opti-MEM^®^ medium was added, and the cells were incubated for 2, 4, 6, and 8 h, respectively. Subsequently, the medium was carefully aspirated, and the cells were gently washed thrice with pre-cooled PBS. The cells were fixed with 4% paraformaldehyde for 10 min at room temperature. After fixation, the cells were washed thrice with pre-cooled PBS and permeabilized with 0.1% Triton X-100 in PBS for 30 min. The cells were washed thrice with pre-cooled PBS for 3 min each. The cells were blocked with a QuickBlock^TM^ Blocking Buffer for Immunol Staining for 15 min at room temperature. After blocking, cells were incubated with an Alexa Fluor^®^ 647 Anti-LAMP1 antibody, diluted 1:200 in QuickBlock^TM^ Primary Antibody Buffer for Immunol Staining, for 2 h at room temperature. The cells were washed again thrice with PBS for 3 min each, followed by nuclear staining with Hoechst 33258 (10 μg/mL) for 10 min. Finally, the cells were washed thrice with pre-cooled PBS for 3 min each, soaked in HBSS buffer, and imaged using a DeltaVision Ultra high-resolution live cell imaging system (Cytiva, Wilmington, DE, USA). Image J software (ImageJ-win64, version 1.54) was used for image analysis.

#### 2.2.7. BMDCs Activation Experiment

Six-to-eight-week-old specific pathogen-free male C57BL/6 mice were purchased from Beijing Viewsolid Biotechnology Co., Ltd. (Beijing, China). All procedures were performed in accordance with the guidelines of the Institutional Animal Care and Use Committee for Animal Experimentation. For BMDC culture, mice were euthanized by cervical dislocation, soaked in 75% ethanol for 2–3 min, and the tibias and femurs were harvested to isolate bone marrow cells. Bone marrow cells were extracted by flushing the shaft of the long bones with a 25 g needle using an RPMI 1640 medium containing 10% FBS and a 1% Penicillin-Streptomycin-Amphotericin B Solution to create a homogeneous suspension. The suspension was strained through 70 mm cell strainers, and the cells were centrifuged at 1000 rpm for 3 min. Red blood cells were lysed with 2 mL of red blood cell lysis buffer for 2–3 min, and the lysis was terminated with 5 mL of an RPMI 1640 complete medium, followed by centrifugation at 1000 rpm for 5 min. The cells were washed with 5 mL of an RPMI 1640 complete medium and centrifuged again at 1000 rpm for 3 min. The supernatant was discarded, and the cells were resuspended in RPMI 1640 complete medium containing 20 ng/mL of Murine GM-CSF. The cell concentration was adjusted to 1 × 10^6^ cells/mL; cells were seeded in 6-well plates at 4 mL/well and cultured at 37 °C with 5% CO_2_ (designated as day 0). On the second and fourth days, half of the culture medium and GM-CSF were replaced with fresh equivalents to maintain optimal growth conditions. Cells were collected via centrifugation on the sixth day.

The collected BMDCs were seeded into 12-well plates at a density of 1 × 10^6^ cells/well. PBS and LNPs (mRNA concentration, 1 μg/mL) were added to each well, followed by a 24 h incubation. After incubation, cells were collected via centrifugation at 350× *g* for 5 min at 4 °C. Cells were resuspended in 100 μL of pre-cooled PBS, and 1 μL of Brilliant Violet 421^TM^ anti-mouse CD11c antibody, APC anti-mouse CD40 antibody, PE anti-mouse CD80 antibody, and 4 μL of Brilliant Violet 510^TM^ anti-mouse CD86 antibody were added, respectively. CD11c was mixed with CD40, CD80, and CD86. To prevent fluorescent dye interactions, single-staining controls for each antibody (CD11c, CD40, CD80, and CD86) were prepared. The cells were incubated on ice for 30 min in the dark and washed thrice with pre-cooled PBS. Three duplicate wells were used for each sample. The cells were resuspended in 200 μL of PBS. Data were acquired using an Attune NxT flow cytometry analyzer (Invitrogen), and the percentage of positively stained cells was determined.

#### 2.2.8. Immunization

Female BALB/c mice (6–8 weeks old) were purchased from Beijing Viewsolid Biotechnology Co., Ltd. (Beijing, China). All experimental procedures involving animals were conducted in strict compliance with the guidelines of the Institutional Animal Care and Use Committee for Animal Experimentation. The mice were immunized three times, 14 days apart, with LNPs (10 μg mRNA per dose) via intramuscular (i.m.) and intranasal (i.n.) routes. Serum samples were collected on days 7, 21, and 35 (i.e., one week after the final immunization) for antibody response analysis. The mice were humanely euthanized one week after the final immunization. Nasal and lung lavage fluids were obtained through three consecutive washes with 200 μL of Dulbecco’s phosphate-buffered saline (D-PBS) for each site, respectively, for antibody response analysis. The major organs (heart, liver, spleen, lungs, and kidneys) were collected for hematoxylin and eosin (H&E) staining to evaluate biosafety.

#### 2.2.9. Enzyme-Linked Immunosorbent Assay (ELISA)

IgG, IgG1, and IgG2a titers against the Omicron BA.4/5 RBD of SARS-CoV-2 were determined using an indirect ELISA. ELISA plates (Corning) were coated overnight at 4 °C with 1 μg/mL of SARS-CoV-2 Omicron BA.4/5 RBD recombinant protein in ELISA Coating Buffer (1×, pH 9.6) and blocked with 2% BSA in PBST (1×). Serum samples were serially diluted threefold and added to each well. Plates were incubated with goat anti-mouse IgG-HRP, IgG1-HRP, or IgG2a-HRP antibodies at 37 °C for 1 h, followed by color development with 3,3′,5,5′-tetramethylbenzidine (TMB) substrates. Reactions were halted with an ELISA Stop Solution, and absorbance was measured at 450 nm using a microplate reader (Varioskan Lux, Thermo Fisher Scientific, Waltham, MA, USA). The endpoint titer was defined as the highest serum dilution, yielding an absorbance 2.1 times greater than background values. If the OD value did not reach 2.1 times the background value, the titer was considered zero, even if it was below the initial dilution. For IgA analysis, lung, nasal wash, and serum samples were serially diluted two-fold, with all other procedures following the same protocol as for IgG analysis.

#### 2.2.10. Hematoxylin and Eosin (H&E) Staining

Excised mouse organs were fixed using a fixative solution, dehydrated with ethanol, made transparent with xylene, and embedded in paraffin to create paraffin sections. Subsequently, the paraffin sections were deparaffinized, stained with hematoxylin, stained with eosin solution, and subjected to a series of dehydration steps. After clarification, the sections were mounted for imaging. Images were captured using a PrimoStar Upright Microscope (Carl Zeiss, Jena, Germany).

#### 2.2.11. Statistical Analysis

The data in this study are presented as the mean-value ± standard error of the mean. All statistical analyses were performed using GraphPad Prism software (version 9.5.0; GraphPad, La Jolla, CA, USA). Statistical significance was determined using a one-way analysis of variance (ANOVA). * *p* < 0.05, ** *p* < 0.01, *** *p* < 0.001, **** *p* < 0.0001, ns indicates no significant difference.

## 3. Results and Discussion

### 3.1. mRNA Identification

Agarose gel electrophoresis was performed to verify the size of the linear DNA, ensuring that it matched the target gene, as depicted in Figure 1C. The electrophoresis band positions for the linear DNA corresponded to the expected sizes of the target RBD plasmid (~3500 bp) and RBD-GFP plasmid (~4300 bp). The absence of additional bands of varying fragment sizes suggests that the sequence integrated into the pUC57 vector was in accordance with the intended sequence and that the plasmid template was completely linearized. Incomplete linearization of the plasmid template may lead to RNA product fragments that are larger than expected, compromising mRNA quality. The mRNA fragment sizes were in concordance with the dimensions of the target gene, with the mRNA sequences of RBD and RBD-GFP measuring approximately 970 and 1700 bp, respectively. Following in vitro transcription and capping, the target bands were distinct, with no observable degradation or dispersion, indicating good mRNA integrity and stability (Figure 1D).

### 3.2. LNP Formulation and Characterization

From the ^1^H-NMR spectrum (Figure 2C,D), the proton peak of CH2 (2.81 ppm) in DMG-PEG2000-Mannose and DMG-PEG2000-Chitosan disappears after modification, indicating the connection between DMG-PEG2000-NHS and chitosan or mannose. The gel permeation chromatography (GPC) results indicate that the molecular weight has increased after modification, with the retention time in the GPC chromatogram shifting forward and a single peak appearing (Figure 2E).

LNPs were prepared according to previously described methods and characterized for particle size, polydispersity index, and zeta potential using dynamic light scattering (Figure 2F). The particle sizes of the LNP formulations were consistently within the 100–200 nm range, with polydispersity indices <0.3, indicating a relatively uniform particle distribution. The optimal LNP size is generally 20–200 nm, which ensures sufficient robustness to withstand fluid flow (e.g., blood and lymph) while allowing LNPs to cross the interstitium [34,35,36]. Upon modification with DMG-PEG2000, the particle size of LNPs exhibited an incremental increase as the molar percentage (mol%) rose from 0.3 mol% to 1.5 mol%, except for 0.5 mol% DMG-PEG2000-CS. The LNPs modified with CS exhibited larger particle sizes than those modified with Man at equivalent molar percentages. LNP self-assembly is promoted by the hydrophilic spatial barrier formed by PEG lipids on the LNP surface through PEG chains [37]. This may be because the molecular weight (MW) of chitosan is larger than that of mannose, and it is presumed that the PEG-lipid was located only at the LNP surface [38], resulting in a slightly larger particle size of the CS-modified LNP formulations. Except for the larger particle size of 1.5 mol% DMG-PEG2000-Man, the particle sizes of DMG-PEG2000-Man LNPs were comparable with those of unmodified DMG-PEG2000 LNPs. The zeta potentials of all formulations were neutral or weakly charged. If the zeta potential fell between −20 and +20 mV, the surface charge was considered weak [39]. The encapsulation efficiencies of all LNP formulations were greater than 85% (Figure 2F). Compared to week 0, after 2 weeks of storage at 4 °C, there was essentially no change in the encapsulation efficiency of the five groups of LNP formulations (Figure S1). At 4 weeks, the encapsulation efficiency of the mRNA-LNP-0.3CS group and the mRNA-LNP-0.3Man group remained essentially unchanged, while the encapsulation efficiency of mRNA-LNP, mRNA-LNP-0.3CS, and mRNA-LNP-0.3Man decreased by 5.48%, 5.17%, and 6.15%, respectively (Figure S1). This indicates that mRNA-LNP, mRNA-LNP-0.3CS, and mRNA-LNP-0.3Man maintain good stability within 2 weeks, and the encapsulation efficiency begins to decline after 4 weeks (Figure S1).

### 3.3. Transfection Efficiency of LNPs in DC2.4 Cells

To verify the transfection efficiency of various LNP formulations in DC2.4 cells, the mRNA of RBD fused with green fluorescent protein was used. The transfection outcomes for the various formulations are shown in Figure 2G,H. It was observed that for LNPs containing the same PEGylated lipid, the transfection efficiency declined as the mol% of the lipid increased from 0.3 mol% to 1.5 mol%. The highest transfection efficiency was observed with the LNP formulation containing 0.3 mol% of the modified DMG-PEG2000. The mean fluorescence intensity results align with the transfection efficacy, with mRNA-LNP, mRNA-LNP-0.3CS, and mRNA-LNP-0.3Man exhibiting average fluorescence intensities of 2369.3 ± 7.23, 2011.7 ± 23.50, and 2513.7 ± 20.98, respectively (Figure 2E). LNP formulations modified with 0.3 mol% CS exhibited slightly lower transfection efficiency compared to those of the 0.3 mol% Man-modified group and 1.5 mol% DMG-PEG2000 group, likely because of the larger particle size of the 0.3 mol% CS-modified group. Based on these findings, the LNP formulation containing 0.3 mol% modified DMG-PEG2000, which demonstrated the highest transfection efficiency, was selected for subsequent studies of cellular uptake and TEM analysis.

### 3.4. Morphological Characterization of LNPs Using TEM

**Figure 3:**
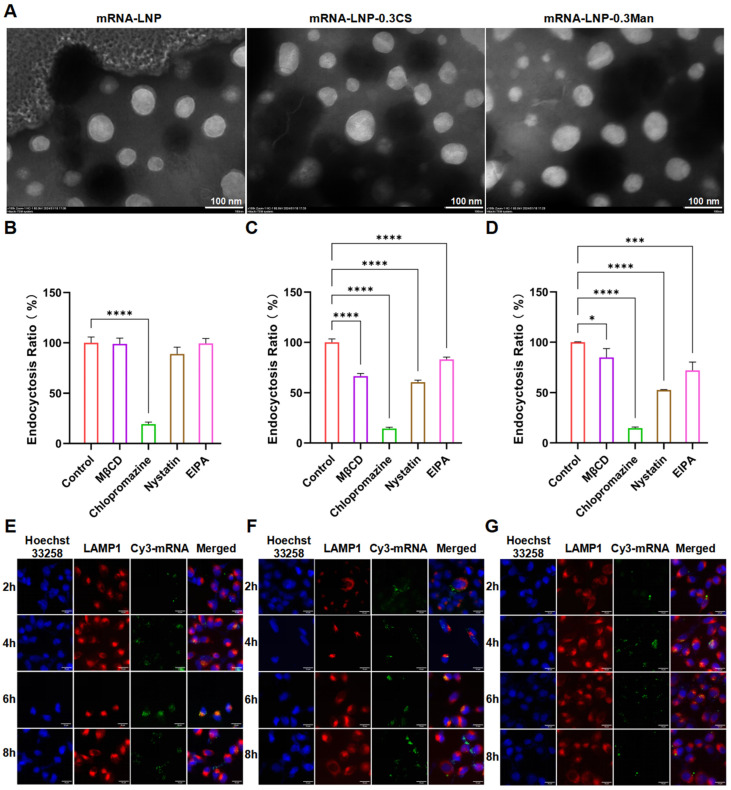
(**A**) Transmission electron microscopy (TEM) images of LNPs (scale bar, 100 nm). (**B**–**D**) Effects of different endocytosis inhibitors on LNP uptake by DC 2.4 cells (**B**) mRNA-LNP; (**C**) mRNA-LNP-0.3CS; (**D**) mRNA-LNP-0.3Man), * *p* < 0.05, *** *p* < 0.001, **** *p* < 0.0001. (**E**–**G**) Co-localization of LNPs with lysosomes in DC 2.4 cells (**E**) mRNA-LNP; (**F**) mRNA-LNP-0.3CS; (**G**) mRNA-LNP-0.3Man; scale bar, 15 μm).

The TEM results demonstrated that the LNPs of different formulations exhibited regular morphology, appearing spherical or spheroidal with a smooth surface, relatively uniform size, and good dispersibility. There were no significant differences in the morphology of LNPs across the different formulations (Figure 3A).

### 3.5. Cellular Uptake

Various inhibitors of cellular uptake pathways were co-incubated with DC 2.4 cells to elucidate the mechanism of LNP uptake. The effects of these inhibitors on DiI-LNP endocytosis are shown in Figure 3B–D. Clathrin, which plays a critical role in transporting biomolecular hormones, neurotransmitters, and membrane proteins, is a key component of clathrin-mediated endocytosis, one of the most widely studied endocytic mechanisms [40,41,42]. Clathrin-dependent uptake can be established by obstructing the active/inactive state of the AP2 complex, leading to its depletion from the plasma membrane, thereby preventing the formation of clathrin-coated pits [43]. Chlorpromazine, an inhibitor of clathrin-mediated endocytosis, prevents AP2 aggregation on the cell surface [44]. Caveolae-mediated endocytosis involves 60–80 nm membrane invaginations that absorb extracellular fluid components [45]. Nystatin, a commonly used inhibitor, sequesters cholesterol from the plasma membrane, destabilizes caveolar formation and impairs caveolae-mediated endocytosis [46]. MβCD depletes cholesterol from the plasma membrane, while nystatin binds to it, further disrupting caveolae. EIPA inhibits macropinocytosis by interfering with Na+/H+ exchangers in the plasma membrane [47]. Chlorpromazine significantly inhibited mRNA-LNP endocytosis, whereas the other inhibitors did not, indicating that mRNA-LNP endocytosis is related to clathrin and is primarily clathrin-mediated (Figure 3B). In the presence of Chlorpromazine, 19.3 ± 2.0% of cells were loaded versus 100.0 ± 5.9% in the control, indicating about an 80.7% decrease in the endocytosis ratio. However, the endocytosis efficiency was not significantly reduced in the presence of the other three inhibitors. In Figure 3C, the decrease in the endocytosis ratios of mRNA-LNP-0.3CS were 85.5 ± 1.3%, 39.5 ± 2.0%, 33.4 ± 2.3%, and 17.0 ± 2.6% in the presence of Chlorpromazine, Nystatin, MβCD and EIPA respectively. This showed the four inhibitors significantly reduced the cellular uptake. In Figure 3D, the decrease in the endocytosis ratios of mRNA-LNP-0.3Man were 15.1 ± 8.8%, 85.3 ± 1.1%, 47.4 ± 0.5%, and 27.8 ± 8.2%, respectively, with the presence of four inhibitors respectively from left to right. All four inhibitors significantly reduced the cellular uptake of mRNA-LNP-0.3CS (Figure 3C) and mRNA-LNP-0.3Man (Figure 3D). The inhibitory effect on mRNA-LNP-0.3CS was ranked as Chlorpromazine > Nystatin > MβCD > EIPA, and for mRNA-LNP-0.3Man as Chlorpromazine > Nystatin > EIPA > MβCD. These results suggest that the uptake of mRNA-LNP-0.3CS and mRNA-LNP-0.3Man may involve multiple pathways, including clathrin-mediated endocytosis, caveolae/lipid raft-mediated endocytosis, and macropinocytosis, with clathrin-mediated endocytosis being the predominant pathway. The involvement of other pathways appeared to vary slightly based on the composition of the modified PEGylated lipids.

### 3.6. Endosome Escape

To investigate the impact of modified PEGylated lipids on endosome escape efficiency, the colocalization and timing of colocalization release between LNPs and lysosomes were examined. Owing to the strong membrane-binding ability of DiI after cellular uptake, it is unsuitable as a fluorescence marker for lysosomal colocalization. Therefore, Cy3-mRNA was selected as the nucleic acid encapsulated within LNPs. The colocalization results of LNPs and lysosomes, observed using the DeltaVision Ultra high-resolution live-cell imaging system, are shown in Figure 3E–G. Additionally, we performed tangent analysis on the co-localization images of LAMP-1 and Cy3-mRNA in each endosome escape figure within the text (Figures S2–S4). According to the results of the tangent analysis, it can be concluded that within the incubation time of 4 h or 6 h, the green fluorescence of mRNA in the three groups of LNPs overlapped with the red fluorescence curve of lysosomes to a relatively high degree. After 8 h, the overlap between the green and red curves was low, indicating that LNPs successfully escaped from endosomes in DC 2.4 cells. These findings also demonstrated that the modified PEGylated lipids at a concentration of 0.3 mol% did not affect the endosome escape efficiency.

### 3.7. BMDCs Activation Experiment

**Figure 4:**
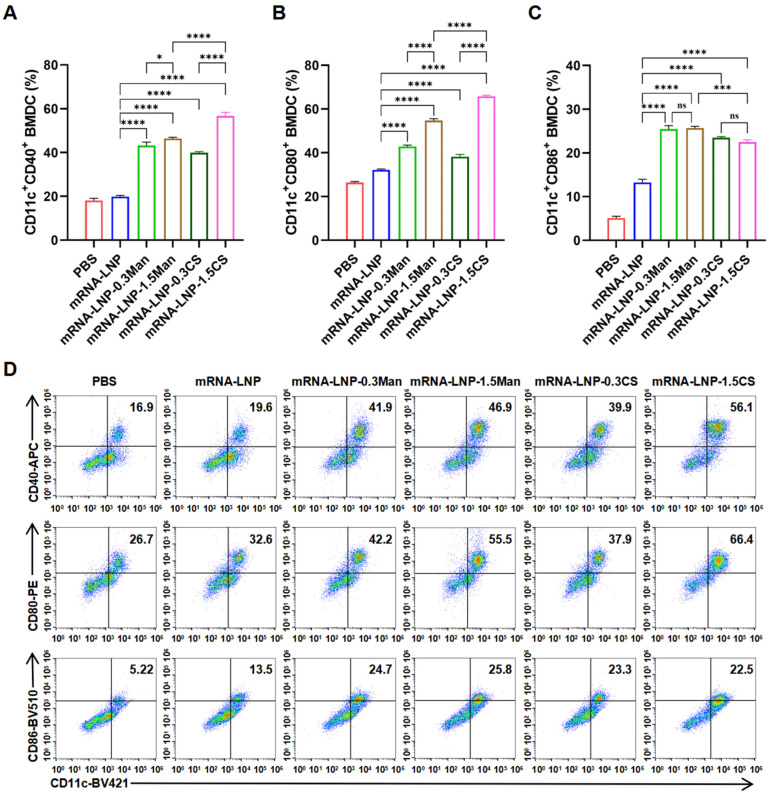
(**A**) Percentage of CD40 determined by flow cytometry (FCM), * *p* < 0.05, **** *p* < 0.0001. (**B**) Percentage of CD80 expression on DCs determined by FCM, **** *p* < 0.0001. (**C**) Percentage of CD86 expression on DCs determined by FCM, *** *p* < 0.001, **** *p* < 0.0001, ns indicates no significant difference. (**D**) Typical FCM figures.

DCs are the most powerful professional antigen-presenting cells (APCs) in the body, playing a crucial role as immune sentinels by initiating T-cell responses (mature DCs can effectively activate initial T cells) and linking innate and adaptive immunity [48,49]. CD40, CD80, and CD86 are co-stimulatory molecules expressed on APCs that serve as markers of DC maturation [50,51]. CS and Man have been shown to enhance the immunogenicity of vaccines, with a theoretical correlation between higher doses and improved immune responses. The study found that the ability of chitosan-modified polysaccharide nanoparticles to activate BMDCs is related to their MW and dosage, and there is a clear dose-response relationship [52]. In this study, we evaluated the activation of BMDCs using modified PEGylated lipids at 1.5 mol% and a group with higher transfection efficiency (0.3 mol%) to assess their ability to activate BMDCs. Various LNP formulations were employed to stimulate BMDCs, and their ability to induce maturation and activation was assessed by quantifying the expression of surface markers CD86, CD80, and CD40 (Figure 4). Stimulation with mRNA-LNP-0.3CS, mRNA-LNP-1.5CS, mRNA-LNP-0.3Man, and mRNA-LNP-1.5Man resulted in significant upregulation of CD86, CD80, and CD40 in BMDCs compared to that in the PBS and mRNA-LNP groups. This indicated that mRNA-LNP-0.3CS, mRNA-LNP-1.5CS, mRNA-LNP-0.3Man, and mRNA-LNP-1.5Man significantly promoted BMDC activation, with a greater activation capacity of mRNA-LNP. Notably, except for CD86, formulations containing 1.5 mol% of modified PEGylated lipids showed higher expression levels than those containing 0.3 mol%, particularly in the mRNA-LNP-1.5CS group, which exhibited the most significant upregulation. This suggests that increasing the concentration of modified PEGylated lipids significantly enhanced BMDC activation, with the mRNA-LNP-1.5CS formulation demonstrating the highest activation capability. Interestingly, this trend contrasts with that observed in the DC2.4 cell transfection efficiency studies. To further validate these findings regarding vaccine efficacy, immune responses in mice were assessed.

### 3.8. Immunization

**Figure 5:**
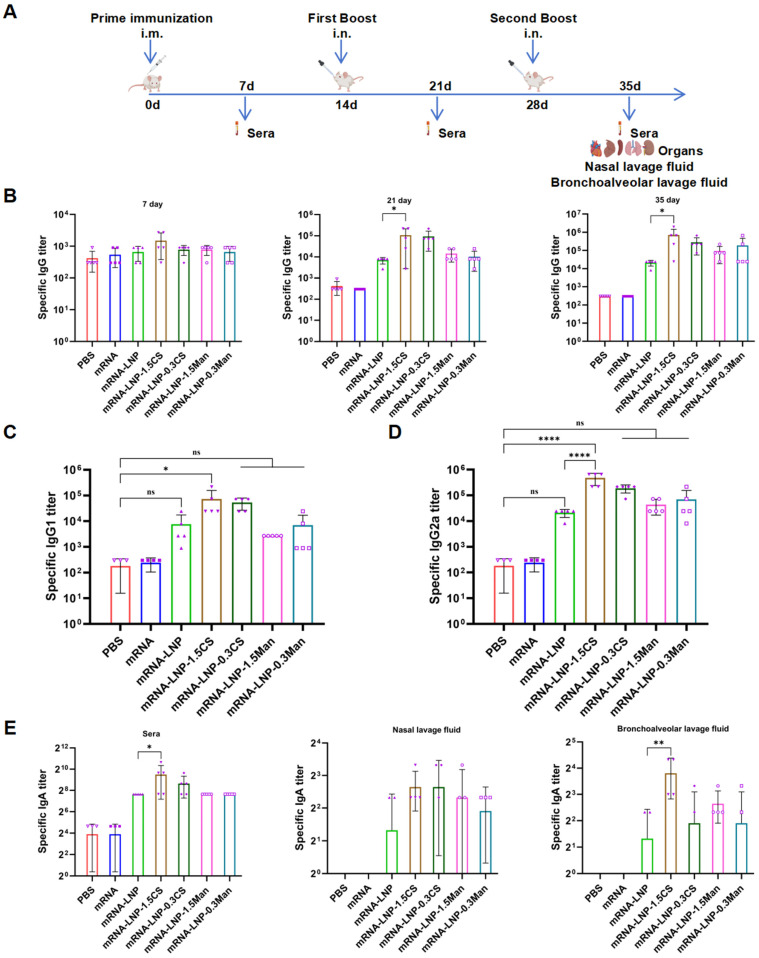
(**A**) Schematic of the vaccination schedule of mice. (**B**) Omicron BA.4/BA.5 RBD-specific IgG titers detected in mouse serum by ELISA on days 7, 21, and 35 (*n* = 5), * *p* < 0.05. (**C**) Omicron BA.4/BA.5 RBD-specific IgG1 subtype titers detected in mouse serum by ELISA on day 35 (*n* = 5), * *p* < 0.05, ns indicates no significant difference. (**D**) Omicron BA.4/BA.5 RBD-specific IgG2a subtype titers detected in mouse serum by ELISA on day 35 (*n* = 5), **** *p* < 0.0001, ns indicates no significant difference. (**E**) Omicron BA.4/BA.5 RBD-specific sIgA titers detected in mouse serum (*n* = 5), nasal lavage fluid (*n* = 4), or bronchoalveolar lavage fluid (*n* = 4) by ELISA on day 35, * *p* < 0.05, ** *p* < 0.001.

PBS, mRNA, and various formulated LNPs were administered thrice to mice via i.m. and i.n. routes at two-week intervals (Figure 5A). Mouse sera were collected at three time points and Omicron BA.4/BA.5 RBD-specific IgG titers were measured (Figure 5B). On the 7th day after immunization, the antibody titer induced by mRNA-LNP-1.5CS was higher than that in the other groups, although there was no significant difference due to the low titer among the groups. By the 21st day post-immunization, both mRNA-LNP-1.5CS and mRNA-LNP-0.3CS groups exhibited higher antibody titers than in the other groups, with the titer in the mRNA-LNP-1.5CS group being significantly higher than that in the mRNA-LNP group. By the 35th day after immunization, the antibody titer ranking for each group was as follows: mRNA-LNP-1.5CS > mRNA-LNP-0.3CS > mRNA-LNP-0.3Man > mRNA-LNP-1.5Man > mRNA-LNP > mRNA > PBS. These results indicated that modified LNPs elicited a stronger IgG antibody response in mice, with CS-modified LNPs showing a superior performance to that of Man-modified LNPs. This may be because the nasal adhesion of mannose is lower than that of chitosan, resulting in a lot of mannose-modified LNP-mRNA being cleared from the nasal cavity [16,53].

Cytokines secreted by Th1 cells mediate isotype switching to IgG2a, whereas those secreted by Th2 cells mediate isotype switching to IgG1 [54]. The ratio of IgG1 to IgG2a represents the balance between cellular (Th1-skewed) and humoral (Th2-skewed) [55]. Therefore, the IgG subclasses (IgG1 and IgG2a) in the sera of the immunized mice were further analyzed (Figure 5C,D). After final immunization, the levels of IgG2a were higher than those of IgG1 across all LNP groups, suggesting a predominantly Th1-skewed immune response. Notably, compared to the PBS group, the levels of IgG1 and IgG2a in the mRNA-LNP-1.5CS group were significantly increased, while other LNP groups showed an increasing trend but without significant differences. The level of IgG2a in the mRNA-LNP-1.5CS group was significantly higher than that in the mRNA-LNP group. These results suggest that intranasal immunization with the mRNA-LNP-1.5CS vaccine can simultaneously promote Th1/Th2 responses, primarily dominated by Th1 immune responses.

Secreted immunoglobulin A (sIgA), the primary antibody involved in local mucosal anti-infection immunity is predominantly found in gastrointestinal fluids, respiratory secretions, and other exocrine fluids. To assess the production of RBD-specific IgA for mucosal immunity, serum, nasal lavage fluid, and bronchoalveolar lavage fluid were obtained from mice after the final vaccination and tested using ELISA (Figure 5E). In intranasally vaccinated mice, the IgA levels in the blood and bronchoalveolar lavage fluid were significantly higher in the mRNA-LNP-1.5CS group than in the mRNA-LNP group. While other modified groups exhibited a trend toward increased IgA levels in the nasal and bronchoalveolar lavage fluids when compared to the mRNA-LNP group, these increases did not reach statistical significance, potentially owing to variability among individual mice or insufficient antibody titers. Notably, IgA titers in the nasal lavage fluid were lower than those in the bronchoalveolar lavage fluid, which may be attributed to the relatively smaller surface area of the nasal mucosa compared to the pulmonary mucosa. These findings suggest that intranasal administration of CS or Man-modified LNP vaccines can induce mucosal immunity, with the mRNA-LNP-1.5CS group demonstrating stronger induction of mucosal immunity.

**Figure 6:**
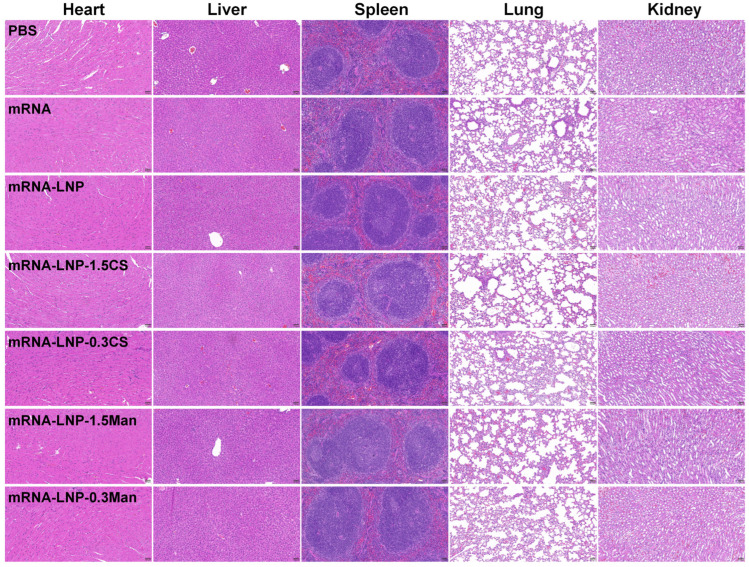
Representative H&E staining images of the main organs at 35 days after immunization (scale bar, 100 μm).

To verify the biosafety of the delivery vectors, various LNPs were evaluated using H&E staining (Figure 6). At 35 days after immunization, no significant damage or inflammation was observed in the major organs of the mRNA and LNP groups compared to those in the PBS group. These findings indicated that mRNA-LNP-1.5CS, mRNA-LNP-0.3CS, mRNA-LNP-1.5Man, mRNA-LNP-0.3Man, and mRNA-LNP demonstrated good bio-tolerance.

After i.m./i.n./i.n. immunization, the modified LNP induced higher IgG and IgA antibody responses in mice, and the CS-modified LNP group showed a superior performance compared with that of the Man-modified LNP group, particularly in the mRNA-LNP-1.5CS group. However, variance analysis results did not reveal significant differences between some groups, potentially because of large intra-group variability. CS may have mucosal adhesions and bind to mucin in the intranasal mucus layer to prolong the retention time of the vaccine in the nasal cavity [53] and enhance immunity. The mRNA-LNP-1.5CS group produced high levels of IgG1 and IgG2a antibodies, promoting Th1/Th2 responses, with a preference for Th1 response. Nasal vaccination against hepatitis B using CS nanocapsules loaded with the TLR7 agonist imiquimod was found to induce high levels of IgG1 and IgG2a antibodies and specific long-term immunity [56].

After 35 days of in vivo immunization, IgG and IgA levels in the blood and bronchoalveolar lavage fluid were significantly higher in the 1.5 mol% group with CS-modified PEGylated lipids than in the unmodified group and slightly higher than those in the 0.3 mol% group. The transfection efficiency of the modified PEGylated lipid molar ratio was contrary to the results of BMDC activation and in vivo immunity. This discrepancy may be related to the combined effect of modified LNPs, facilitating cellular entry through caveolae/lipid rafts, clathrin, macropinocytosis, and other pathways. This may also be attributed to physiological barriers in the mucosal structure that affect vaccine antigen uptake.

## 4. Conclusions

In this study, we successfully developed a nasal mucosal immune mRNA vaccine. When administered i.m./i.n./i.n. to BALB/c mice, the modified PEGylated lipid LNP vaccine induced relatively high levels of IgG and IgA compared with that of unmodified LNPs, with the mRNA-LNP-1.5CS group showing a significant increase. The mRNA-LNP-1.5CS produced high levels of IgG1 and IgG2a antibodies, promoting Th1/Th2 responses, with a preference towards Th1 responses. Notably, CS-modified LNPs elicited a more robust immune response than that with Man-modification. Influenced by the nasal mucosal environment, adhesive materials (e.g., CS) may promote antigen uptake by the nasal mucosa more effectively than receptor-targeting materials (e.g., Man). In contrast, the mRNA-LNP-1.5CS formulation elicited a strong mucosal immune response as well as systemic cellular and humoral immune responses. Our research results also confirmed that chitosan is a material particularly suitable for mucosal drug delivery [57]. Chitosan-based nanocarriers (such as polymeric nanoparticles, liposomes, dendrimers, microspheres, nanoemulsions, solid lipid nanoparticles, and carbon nanotubes)are widely used in the field of local drug delivery [58,59,60], and the most reported is the delivery of siRNA nucleic acid drugs [61]. With the epidemic of COVID-19 and the launch of LNP-mRNA drugs. There is an urgent need to develop LNP-mRNA for nasal administration. Taking into account the significant adhesion function of chitosan. We innovatively modified chitosan on PEG to prepare CS-modified-LNP-mRNA to respond to Omicron. The result is obvious: mRNA-LNP-1.5CS is a safe, effective, and practical mucosal vaccine candidate. One limitation of this study is the complexity of the in vivo and intranasal environments, which are influenced by numerous factors. The immune effects of CS- and Man-modified PEGylated lipids at varying molar ratios did not align with the in vitro transfection rules. Discrepancies between in vivo and in vitro outcomes were not explored in detail in this study, suggesting the need for further investigation.

## Figures and Tables

**Table 1:** Molar ratio of different lipid components and other procedural parameters.

Formulation	SM-102 (mol%)	DSPC (mol%)	Cholesterol (mol%)	DMG-PEG2000 (mol%)	DMG-PEG2000-Chitosan (mol%)	DMG-PEG2000-Mannose (mol%)	N/P	Total Flow (mL/min)
mRNA-LNP	50	10	38.5	1.5	0	0	6	8
mRNA-LNP-1.5CS	50	10	38.5	0	1.5	0	6	12
mRNA-LNP-1.0CS	50	10	38.5	0.5	1.0	0	6	12
mRNA-LNP-0.5CS	50	10	38.5	1.0	0.5	0	6	12
mRNA-LNP-0.3CS	50	10	38.5	1.2	0.3	0	6	12
mRNA-LNP-1.5Man	50	10	38.5	0	0	1.5	6	16
mRNA-LNP-1.0Man	50	10	38.5	0.5	0	1.0	6	16
mRNA-LNP-0.5Man	50	10	38.5	1.0	0	0.5	6	16
mRNA-LNP-0.3Man	50	10	38.5	1.2	0	0.3	6	16

**Table 2:** Function and Concentration of Endocytosis Inhibitors.

Inhibitor	Function	Concentration
Methyl-β-cyclodextrin (MβCD)	Inhibition of caveolae/lipid raft mediated endocytosis by cholesterol depletion	10 μg/mL
Chlorpromazine	Inhibitor of clathrin	10 μg/mL
Nystatin	Inhibitor of caveolae/lipid raft	25 μg/mL
Amiloride (EIPA)	Inhibitor of macropinocytosis	15 μg/mL

## Data Availability

Data are contained within the article.

## Supplementary Materials

The following supporting information can be downloaded at https://www.mdpi.com/article/10.3390/pharmaceutics16111423/s1, Figure S1. Encapsulation efficiency results of different LNPs under 4 °C conditions. Figure S2. Co-localization of mRNA-LNP with lysosomes in DC 2.4 cells (15 μm). The data graph in the far right column is the co-localization analysis of lysosomes and Cy3-mRNA corresponding to the white line scan image in Enlarge. Figure S3. Co-localization of mRNA-LNP-0.3CS with lysosomes in DC 2.4 cells (15 μm). The data graph in the far right column is the co-localization analysis of lysosomes and Cy3-mRNA corresponding to the white line scan image in Enlarge. Figure S4. Co-localization of mRNA-LNP-0.3Man with lysosomes in DC 2.4 cells (15 μm). The data graph in the far right column is the co-localization analysis of lysosomes and Cy3-mRNA corresponding to the white line scan image in Enlarge.
