# Supplementary material for: Modified PEG-Lipids Enhance the Nasal Mucosal Immune Capacity of Lipid Nanoparticle mRNA Vaccines
Source: Pharmaceutics. 2024 Nov 7;16(11):1423. doi: 10.3390/pharmaceutics16111423 (PMC11597600; doi:10.3390/pharmaceutics16111423)
Supplement: Supplementary file 1 [file pharmaceutics-16-01423-s001.zip › pharmaceutics-3252171-supplementary.pdf]

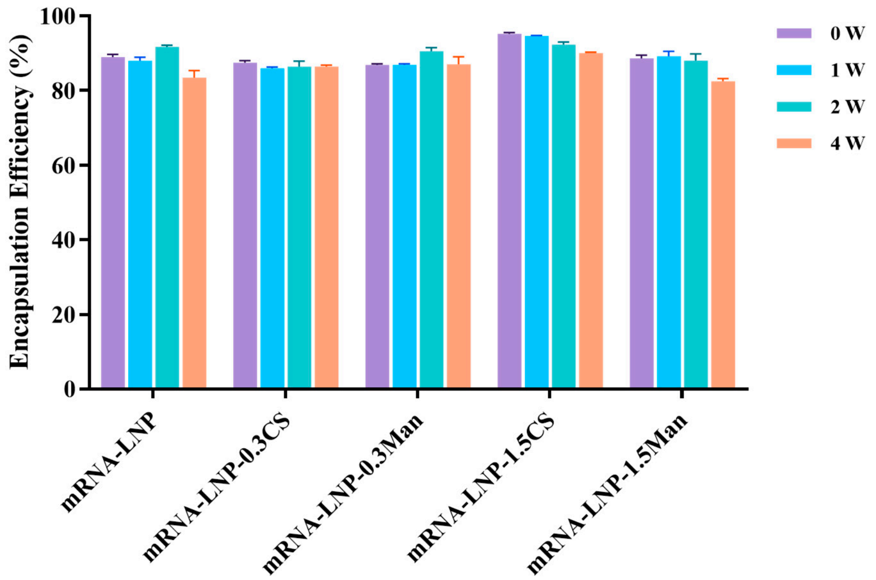

Figure S1. Encapsulation efficiency results of different LNPs under 4°C conditions.

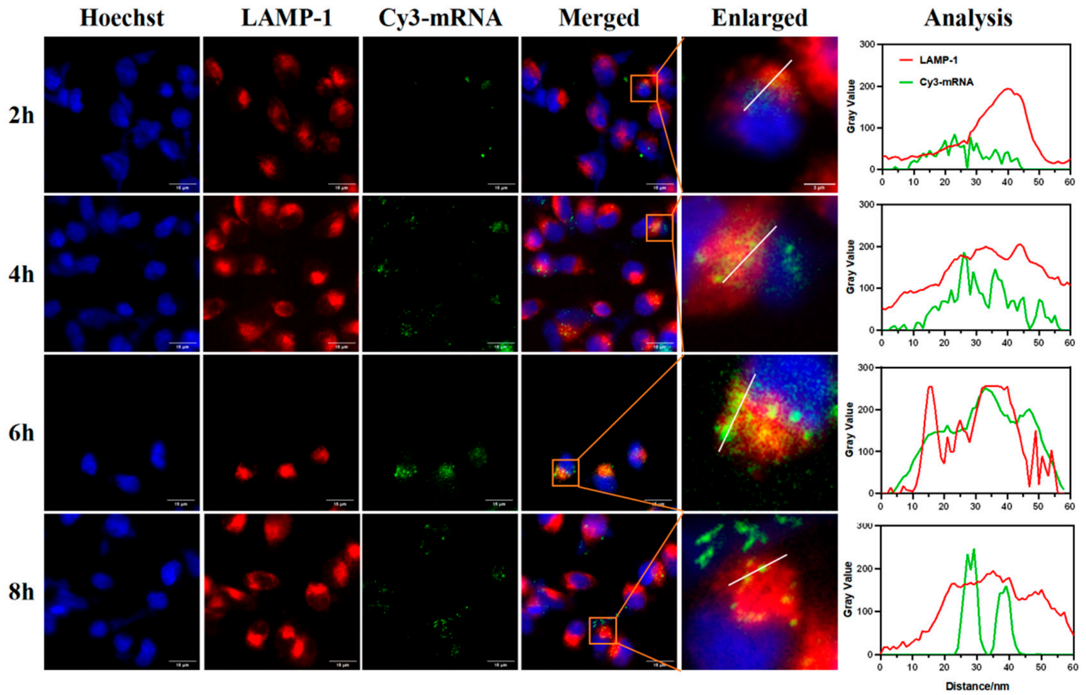

Figure S2. Co-localization of mRNA-LNP with lysosomes in DC 2.4 cells (15 μm). The data graph in the far right column is the co-localization analysis of lysosomes and Cy3-mRNA corresponding to the white line scan image in Enlarge.

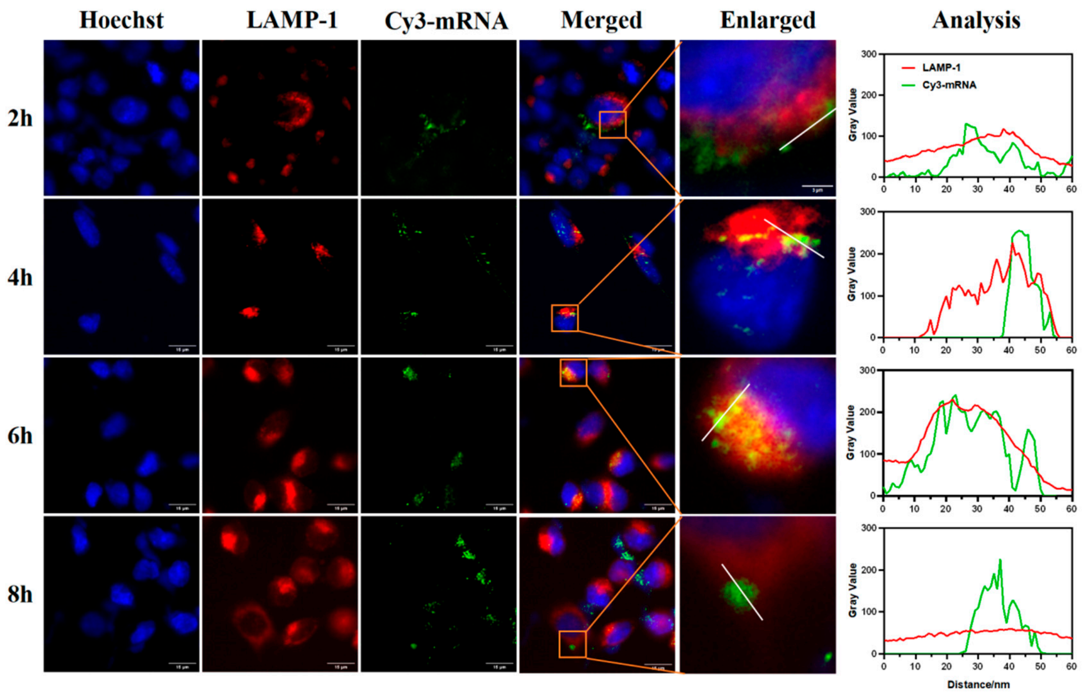

Figure S3. Co-localization of mRNA-LNP-0.3CS with lysosomes in DC 2.4 cells (15 μm). The data graph in the far right column is the co-localization analysis of lysosomes and Cy3-mRNA corresponding to the white line scan image in Enlarge.

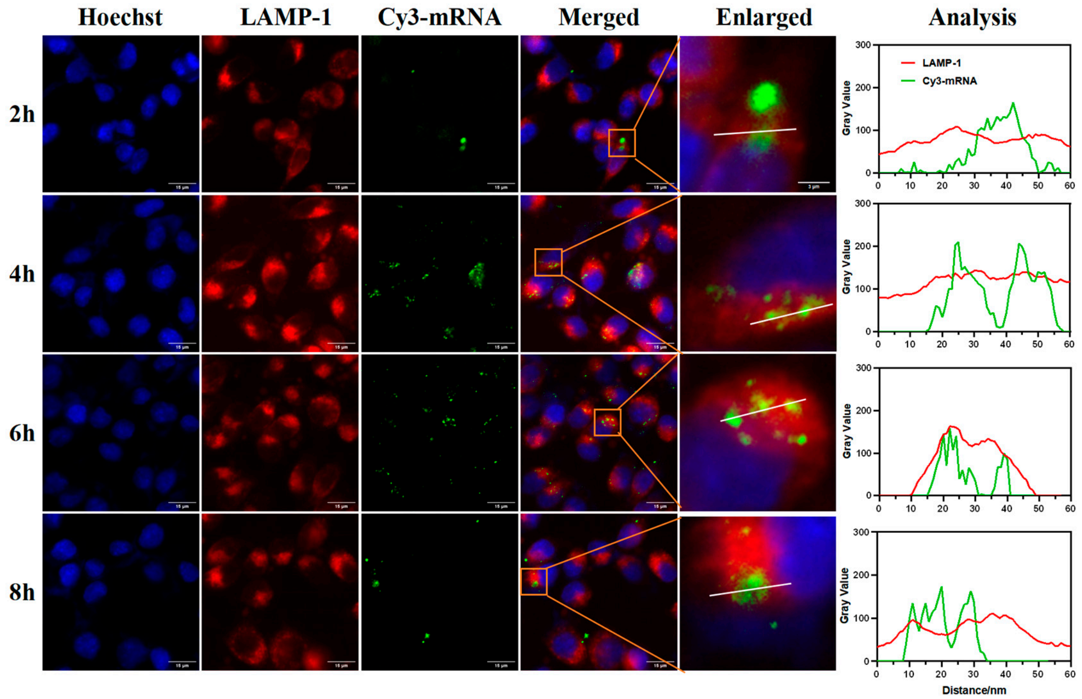

Figure S4. Co-localization of mRNA-LNP-0.3Man with lysosomes in DC 2.4 cells (15 μm). The data graph in the far right column is the co-localization analysis of lysosomes and Cy3-mRNA corresponding to the white line scan image in Enlarge.
